# Metabolomics Analysis of Different Quinoa Cultivars Based on UPLC-ZenoTOF-MS/MS and Investigation into Their Antioxidant Characteristics

**DOI:** 10.3390/plants13020240

**Published:** 2024-01-15

**Authors:** Shufang Wang, Guannan Liu, Chong Xie, You Zhou, Runqiang Yang, Jirong Wu, Jianhong Xu, Kang Tu

**Affiliations:** 1 College of Food Science and Technology, Whole Grain Food Engineering Research Center, Nanjing Agricultural University, Nanjing 210095, China; wangshufang202301@163.com (S.W.); 2022208029@stu.njau.edu.cn (G.L.); xiechong@njau.edu.cn (C.X.); yangrq@njau.edu.cn (R.Y.); 2 Jiangsu Key Laboratory for Food Quality and Safety-State Key Laboratory Cultivation Base, Ministry of Science and Technology/Key Laboratory for Control Technology and Standard for Agro-Product Safety and Quality, Ministry of Agriculture and Rural Affairs/Key Laboratory for Agro-Product Safety Risk Evaluation (Nanjing), Ministry of Agriculture and Rural Affairs/Collaborative Innovation Center for Modern Grain Circulation and Safety/Institute of Food Safety and Nutrition, Jiangsu Academy of Agricultural Sciences, Nanjing 210014, China; zhouyouox@163.com (Y.Z.); yangzhouwjr@126.com (J.W.)

**Keywords:** quinoa, metabolomics, variance analysis, antioxidant properties, UPLC-ZenoTOF-MS/MS

## Abstract

In recent years, quinoa, as a nutritious and sustainable food material, has gained increasing popularity worldwide. To investigate the diversity of nutritional characteristics among different quinoa cultivars and explore their potential health benefits, metabolites of five quinoa cultivars (QL-1, SJ-1, SJ-2, KL-1 and KL-2) were compared by non-targeted metabolomics analysis based on UPLC-ZenoTOF-MS/MS in this study. A total of 248 metabolites across 13 categories were identified. Although the metabolite compositions were generally similar among the different quinoa cultivars, significant variations existed in their respective metabolite contents. Among the identified metabolites, amino acids/peptides, nucleosides, saponins and phenolic acids were the most abundant. Notably, SJ-1 exhibited the most distinct metabolite profile when compared to the other cultivars. Amino acids/peptides and nucleosides were found to be crucial factors contributing to the unique metabolite profile of SJ-1. Collectively, these aforementioned metabolites accounted for a substantial 60% of the total metabolites observed in each quinoa variety. Additionally, a correlation between the DPPH radical scavenging activity and the free phenolic content of quinoa was observed. Variations in phenolic content resulted in different antioxidant capacities among the quinoa cultivars, and SJ-1 exhibited lower phenolic levels and weaker antioxidant activity than the others. These results can provide important information for the development of quinoa resources.

## 1. Introduction

Plant-based foods have gained increasing attention all over the world due to growing concerns about the environmental, ethical, and health impacts associated with animal-sourced foods [1]. Plant seeds are important food ingredients because they provide not only essential nutrients like proteins, lipids, and carbohydrates, but also various small molecular substances that play important roles in maintaining human well-being [2]. For instance, some phytochemicals that are widely found in plant seeds, such as phenolic compounds and carotenoids, are well known for their various health-promoting properties, including antioxidant and anti-inflammatory effects [2]. In recent years, more and more research has focused on the antioxidant properties of phytochemicals in plant seeds [3,4].

Quinoa (*Chenopodium quinoa* Willd.) is often referred to as a pseudocereal because it belongs to dicotyledonous plants, but its seed has similar nutritional and functional characteristics with the grains of monocotyledonous plants, such as millet and rice [5]. In recent decades, quinoa has gained significant popularity worldwide because of its outstanding nutritional value and versatile functional properties.

Generally, quinoa has a lower starch content than grains, and a considerable proportion of starch in quinoa is indigestible starch, which makes quinoa a low-glycemic-index (GI) food [6]. Due to the small size of quinoa starch granules, it is suitable for the preparation of Pickering emulsions [7]. The excellent water retention and gelatinity of quinoa starch can improve the quality of gluten-free bread and help prepare biodegradable membranes [8]. In addition, quinoa contains a high level of protein and can exert some beneficial effects as a source of bioactive peptides [9], as well as a superior gel ability when compared with other plant proteins [10]. Quinoa protein is easier to digest than other grain-based proteins. Gullon et al. analyzed the in vitro digestibility of quinoa protein under various enzymatic hydrolysis systems, and showed that the in vitro digestibility of quinoa protein was ca. 78%, which was higher than that of wheat protein (ca. 55%) [11]. Quinoa also contains more soluble dietary fiber than other plant grains, including inulin, fructooligosaccharides, and galacto-oligosaccharides, which promote the production of short-chain fatty acids (acetic acid, butyric acid and propionic acid) by microorganisms; has a lower intestinal pH; and increases the formation of favorable bacteria [12]. In addition, quinoa contains abundant secondary metabolites, including phenolic acids, flavonoids and other antioxidants, which have been shown to be beneficial to human health [13]. It has been reported that quinoa contains more phenolic substances than most grains and legumes [14]. The regular consumption of polyphenol-containing diets can help prevent cancer, cardiovascular disease, diabetes, osteoporosis, and neurodegenerative diseases [15].

Different cultivars or growing areas have a significant influence on the nutritional characteristics of quinoa, especially in terms of secondary metabolites [16]. Therefore, a comparative analysis of bioactive small-molecule metabolites in quinoa from different cultivars and growing locations is essential to explore their nutritional potentials and the development of quinoa-based foods. Qinghai province is located in the northeastern part of the Tibetan Plateau, and has become a key area for quinoa cultivation in China due to its suitable climate and growing conditions. In the present study, the composition and content of small-molecule metabolites in five quinoa cultivars widely cultivated in Qinghai province were investigated. Meanwhile, their antioxidant abilities and contents of related substances were compared.

## 2. Results

### 2.1. Multivariate Statistical Analysis

The total ion chromatograms of the quality control (QC) and the five samples are shown in Figure S1A. A hierarchical cluster dendritic diagram was employed to explore the relationships and differences between the metabolites of the different quinoa cultivars (Figure S1B). The metabolite composition of SJ-1 exhibited a distinct pattern when compared to the other quinoa cultivars. Principal component analysis (PCA) and partial-least-squares discriminant analysis (PLS-DA) were also conducted to clarify the metabolite differences between the different quinoa cultivars. The first two main principal components (PC1 and PC2) from PCA accounted for 31.4% and 17.1% of total PCA, respectively (Figure S1C), and the samples were well separated. The metabolites of SJ-1 also differed greatly from other samples in both PCA and PLS-DA (Figure S1D). Meanwhile, the metabolites of SJ-2 and QL-1 appeared to display a relatively high degree of similarity, and the metabolite compositions of KL-1 and KL-2 exhibited a strong tendency to cluster closely together, which was consistent with the classification in the hierarchical cluster dendrogram.

### 2.2. Metabolite Composition Analysis

**Figure 1:**
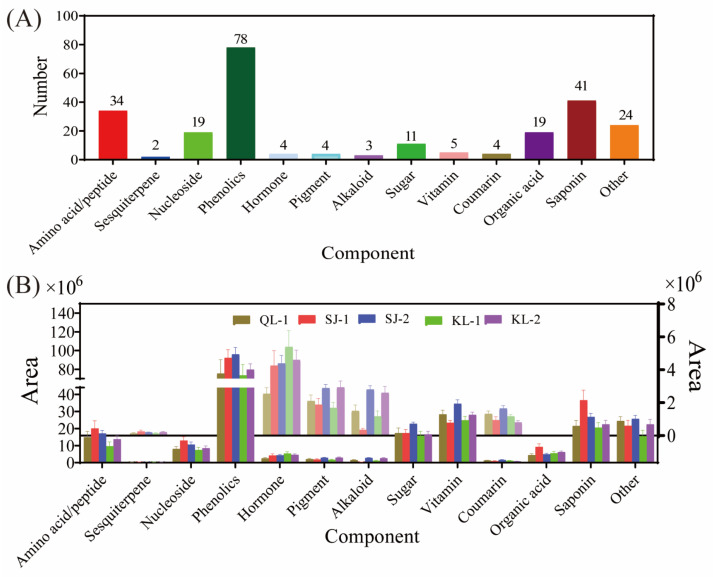
Metabolite composition of different cultivars of quinoa. The number of metabolic species detected (**A**). The area of different species of metabolites in different cultivars of quinoa (**B**).

In total, 13 categories (namely amino acids/peptides, sesquiterpenes, nucleosides, phenolics, hormones, pigments, alkaloids, sugars, vitamins, coumarins, organic acids, saponins and others) and 248 kinds of metabolites were detected in the five quinoa cultivars (Figure 1A). There were 78 kinds of phenolics detected, which was the highest among the 13 categories, followed by saponins (41 kinds), amino acids/peptides (34 kinds), organic acids (19 kinds) and nucleosides (19 kinds). The numbers of metabolites in other categories were less than 15. However, 24 metabolites were classified under the category of other. As shown in Figure 1B, the relative content of phenolics was the highest within all the cultivars. After phenolics, vitamins exhibited the second-highest level of their relative content, followed by saponins, sugars and amino acids. The compositions of detected metabolites in different categories in quinoa are shown in Table S1.

**Figure 2:**
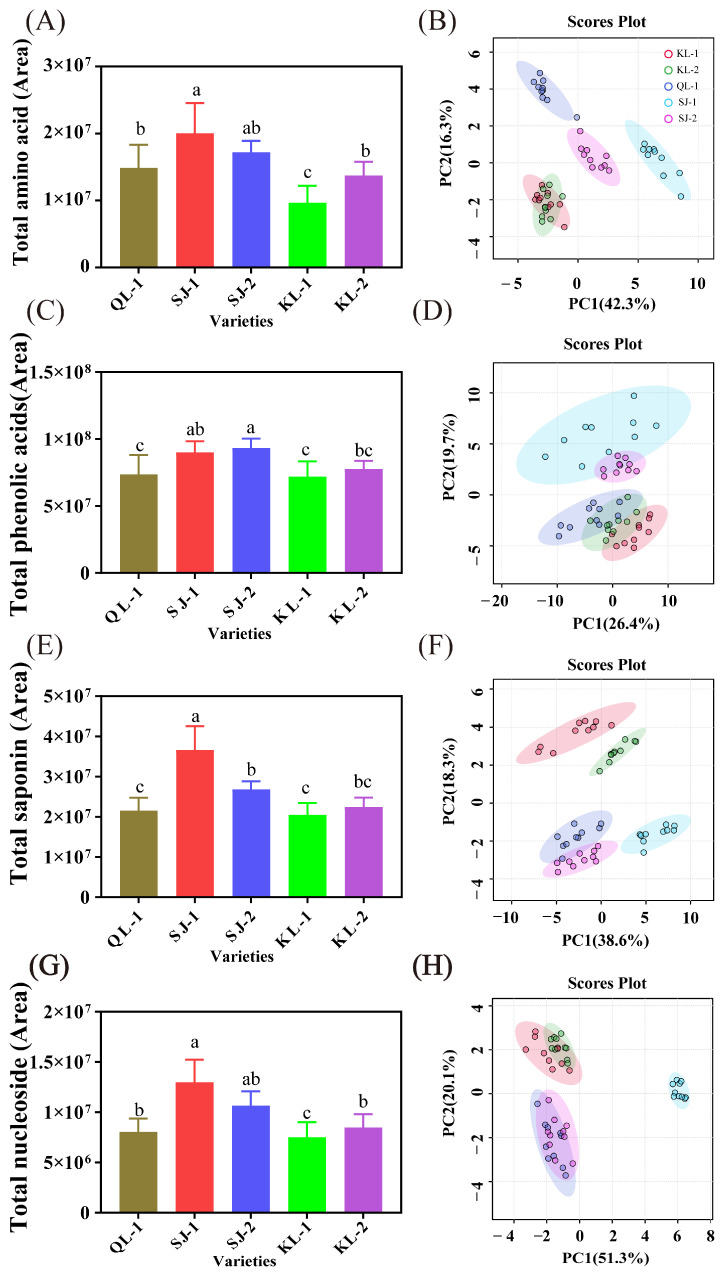
The area of total amino acids (**A**), phenolic acids (**C**), saponins (**E**) and nucleosides (**G**) and PCA analysis of individual metabolites (**B**,**D**,**F**,**H**). Bars with different letters indicate significant differences at *p* < 0.05 for each index.

The relative content of four groups of metabolites (amino acids/peptides, phenolic acids, saponins and nucleosides) and the PCA analysis of individual metabolites are shown in Figure 2, and loading plots as well as the hierarchical clustering heatmap are shown in the Supplementary Materials (Figures S2–S9). SJ-1 had the highest relative content of amino acids/peptides (Figure 2A), saponins (Figure 2E), and nucleosides (Figure 2G) among all the cultivars. SJ-1 and SJ-2 had a similar level of phenolic acids and there were no significant differences among KL-1, KL-2, and QL-1 (Figure 2C). KL-1 had the lowest levels of these four metabolites among all cultivars.

In the PCA analysis of four metabolites, SJ-1 showed significant differences in amino acids/peptides and saponins from other cultivars in terms of the PC1 (Figure 2B,H). In the PCA analysis of phenolic acids, a distinct clustering pattern emerged, with SJ-1 and SJ-2 forming a distinct group, while the remaining three cultivars clustered together (Figure 2D). In terms of saponin, a distinct group was formed between QL-1 and SJ-2 (Figure 2F).

### 2.3. Analysis of Differences in Different Metabolites

#### 2.3.1. Amino Acids/Peptides

**Figure 3:**
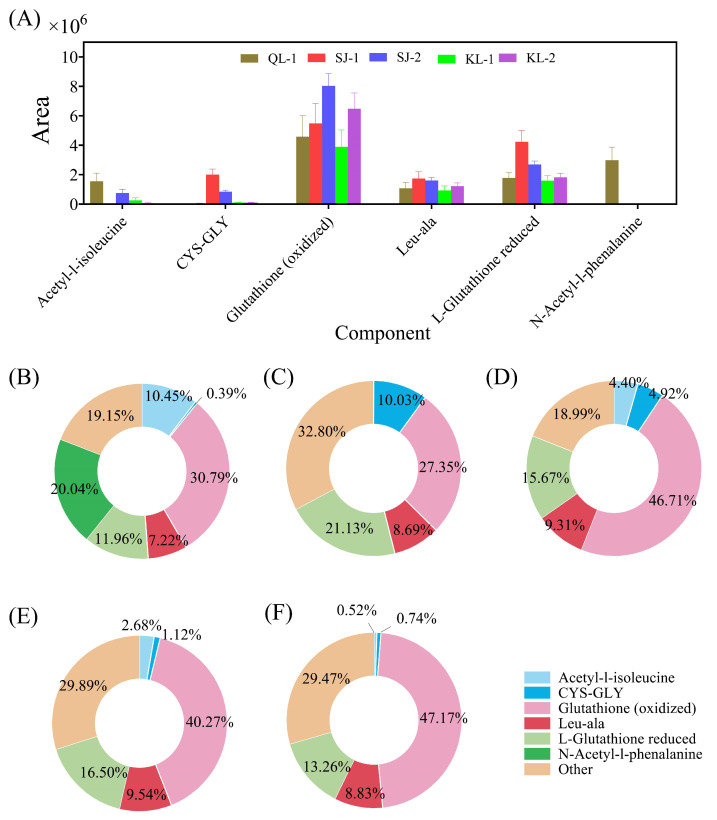
The relative contents of amino acids/peptides in different cultivars of quinoa (**A**) and their ratios in different quinoa cultivars: QL-1 (**B**), SJ-1 (**C**), SJ-2 (**D**), KL-1 (**E**) and KL-2 (**F**).

The area of the six amino acids/peptides accounting for more than 50% of all amino acids are shown in Figure 3A. Glutathione (oxidized) was the most abundant type in these cultivars of quinoa and SJ-2 had the highest amount of Glutathione (oxidized). However, the ratios of Glutathione (oxidized) in all amino acids among cultivars varied from 27.34% to 47.14% (Figure 3B). N-acetyl-1-phenalanine was detected only in QL-1 and acetyl-1-isoleucine was not detected in SJ-1.

#### 2.3.2. Phenolics

**Figure 4:**
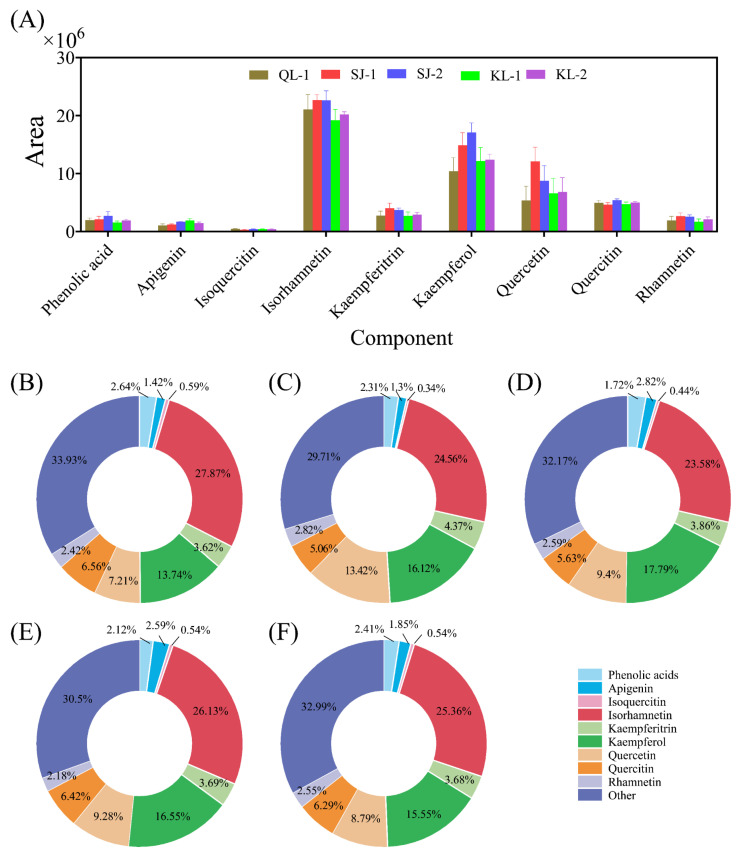
The area of main phenolic acids in different cultivars of quinoa (**A**). Relative monomer content of quinoa phenolic acids of different cultivars: QL-1 (**B**), SJ-1 (**C**), SJ-2 (**D**), KL-1 (**E**) and KL-2 (**F**).

The areas of phenolics of a relatively high content in quinoa are shown in Figure 4. Isorhamnetin, kaempferol and quercetin were three main phenolics present in the five cultivars of quinoa and constituted for ca. 50% of the total areas. Generally, the contents of these phenolics in the SJ cultivars were higher than those of other cultivars. About 30% of the relative monomer contents were classified into the category of others.

#### 2.3.3. Saponins

**Figure 5:**
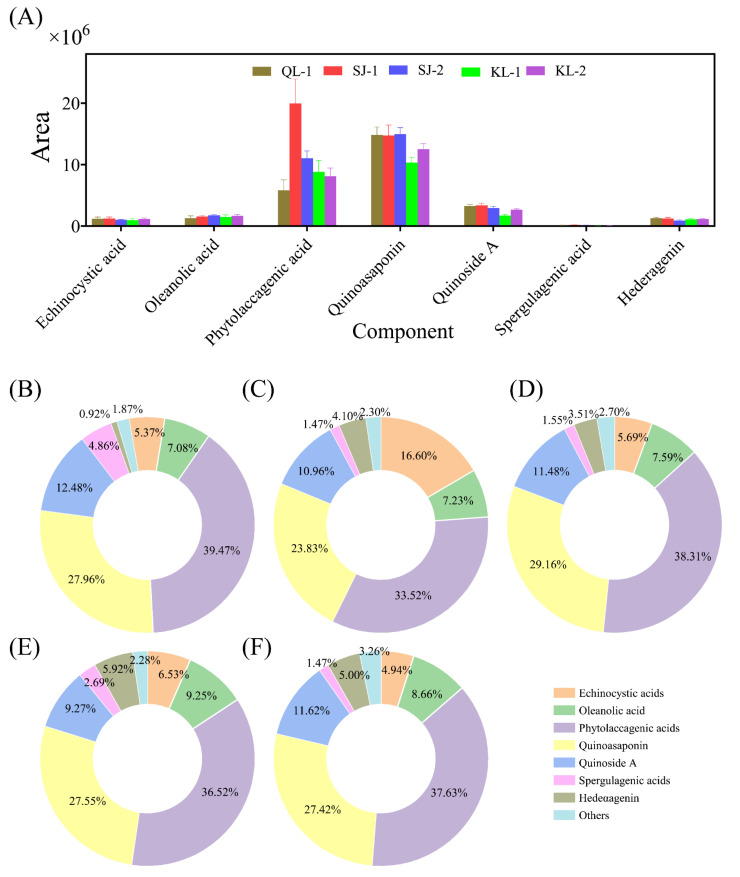
The area of the main saponins in different cultivars of quinoa (**A**). Relative monomer content of quinoa saponins of different cultivars: QL-1 (**B**), SJ-1 (**C**), SJ-2 (**D**), KL-1 (**E**) and KL-2 (**F**).

The area of saponins with a higher content in quinoa is shown in Figure 5A. The relative content of the saponin monomers of all cultivars of quinoa is shown in Figure 5B–F. The profile of saponin monomers detected were similar among the different cultivars of quinoa. The highest contents of saponin monomers consisted of phytolaccagenic acids or quinoasaponin, but their contents varied among the cultivars.

#### 2.3.4. Nucleosides

**Figure 6:**
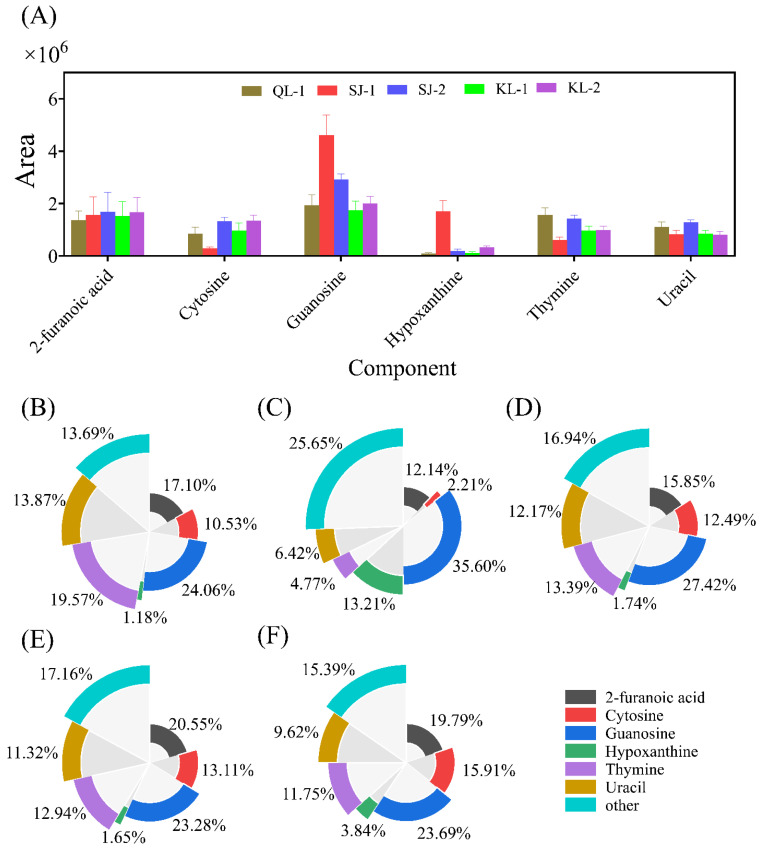
The area of main nucleosides in different cultivars of quinoa (**A**). Relative monomer content of quinoa nucleosides of different cultivars: QL-1 (**B**), SJ-1 (**C**), SJ-2 (**D**), KL-1 (**E**) and KL-2 (**F**).

A total of 19 different nucleoside monomers were detected in quinoa seeds using a non-targeted metabolomics approach. The areas of saponins with high levels are shown in Figure 6A. The nucleoside composition of SJ-1 is the most special, which had a significantly higher levels of guanosine and hypoxanthine but lower levels of cytosine and thymine. The relative contents of the nucleoside monomers of all cultivars are shown in Figure 6B–F. The monomer types detected with a higher nucleoside content were similar in different cultivars of quinoa.

### 2.4. Target Verification of Major Antioxidants and Analysis of Antioxidant Capacity

#### 2.4.1. Total Phenolic Content

**Figure 7:**
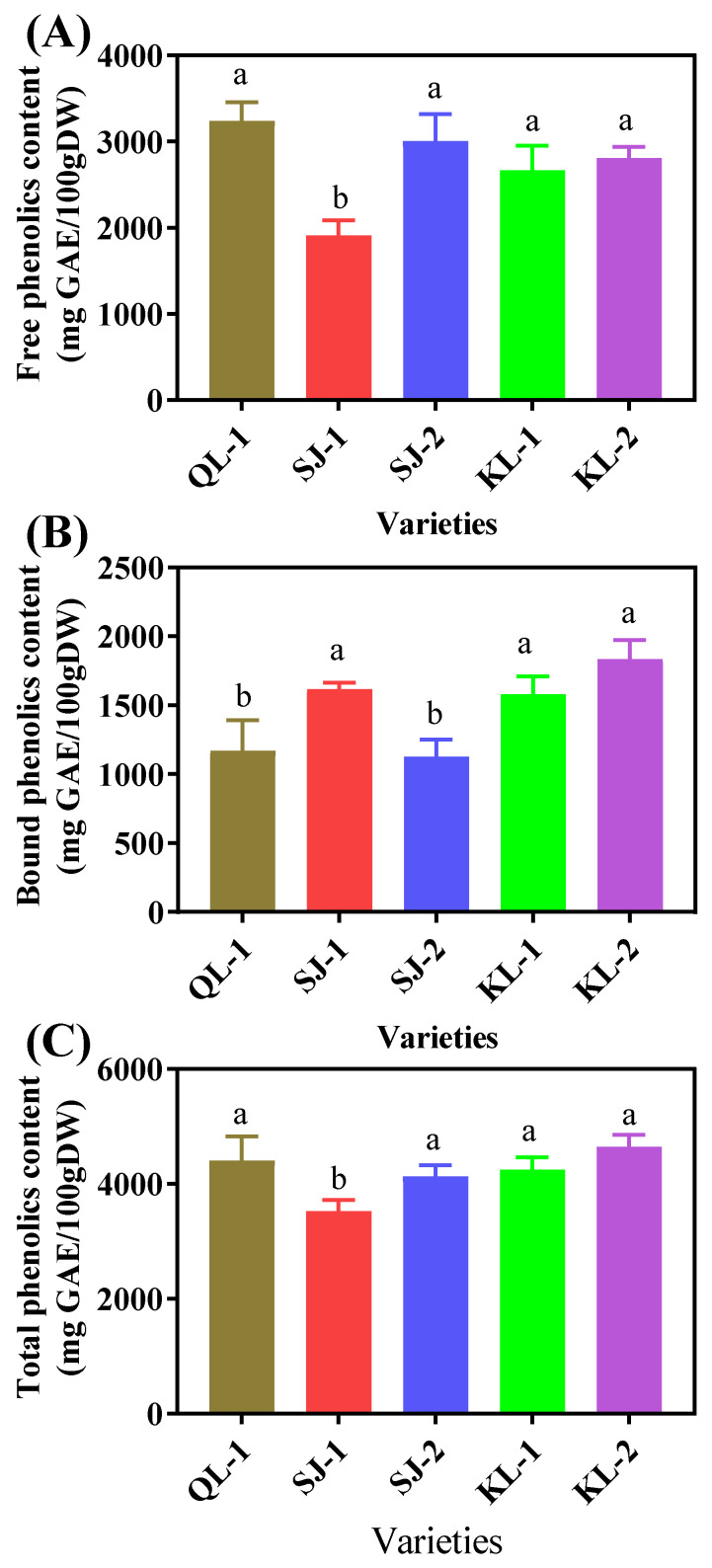
Free phenolic (**A**), bound phenolic (**B**) and total phenolic (**C**) content of different cultivars of quinoa. Bars with different letters indicate significant differences at *p* < 0.05 for each index.

SJ-1 had the lowest content of free phenolics and there were no significant differences among the other four cultivars (Figure 7A). However, QL-1 and SJ-2 had the lowest contents of bound phenolics (Figure 7B). The trend of the total phenolic contents in the five quinoa cultivars was similar to that of the free phenolics (Figure 7C).

#### 2.4.2. Quantification of Phenolic Compounds

Nine kinds of phenolic acids were detected in quinoa, namely gallic acid, protocatechuic acid, *p*-hydroxybenzoic acid, vanillic acid, caffeic acid, syringic acid, *p*-coumaric acid, ferulic acid and sinapinic acid. Ferulic acid was detected at high levels in the bound state rather than in the free state. Caffeic acid was detected at high levels in the free state rather than in the bound state. The rest of the phenolic acids were at comparable levels in the bound and free states.

#### 2.4.3. Antioxidant Capacity

**Figure 8:**
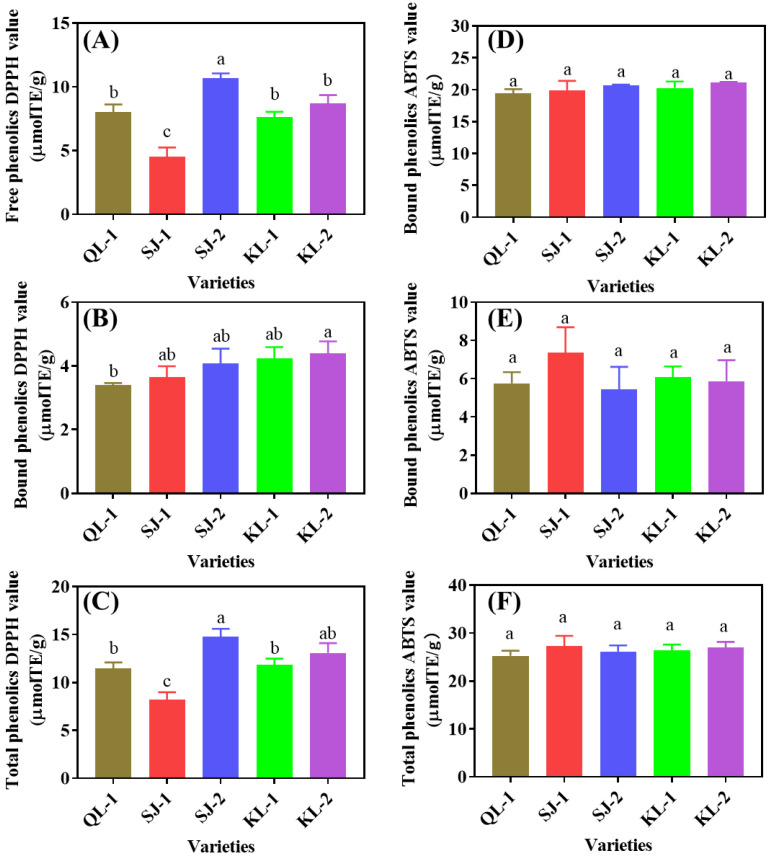
DPPH (**A**–**C**) and ABTS (**D**–**F**) radical scavenging of phenolic substances in different cultivars of quinoa. Bars with different letters indicate significant differences, at *p* < 0.05 for each index.

The 1,1-Diphenyl-2-picrylhydrazyl free radical (DPPH) and 2,2′-Azinobis-(3-ethylbenzthiazoline-6-sulphonate (ABTS) radical-scavenging capacity reflects the antioxidant capacity of different cultivars of quinoa (Figure 8). SJ-2 and SJ-1 showed the highest and lowest DPPH radical-scavenging values among the five cultivars in the free and total phenolics, respectively. However, QL-1 had the lowest DPPH radical-scavenging value of bound phenolics. No significant difference in ABTS radical scavenging was detected among cultivars, no matter whether in free, bound or total phenolics (*p* < 0.05).

## 3. Discussion

Quinoa, recommended by the United Nations as a highly nutritious food for humans, is renowned for its health benefits and excellent nutritional profile [17]. Although quinoa is a native Andean plant, it has spread to other continents. However, the composition of its nutrients, especially the profile of small metabolites in various cultivation areas, remains to be thoroughly investigated. In the present research, metabolites in five cultivars of quinoa cultivated in Qinghai Province, China, were determined by a UHPLC-Q-Orbitrap MS/MS-based untargeted metabolomics approach. A distinct variety in metabolites has been observed among five quinoa cultivars through analysis (Figure S1) and the main factors contributing to these differences, as determined by PCA analysis, were nucleosides, amino acids/peptides, saponins, and phenolic acids.

Amino acids are the basic building blocks of proteins and play an important role in human health [18]. The nutritional value of protein depends on the composition of amino acids. Generally speaking, quinoa has a well-balanced amino acid profile when compared with other plant seeds, such as beans and wheat, which were found to be limited by methionine and lysine, respectively [2,19]. Glutathione is a tripeptide composed of glutamic acid, cysteine, and glycine, which plays an important role in the non-enzymatic antioxidant defense system of plants [20]. The contents of glutathione were found to be the highest in amino acids/peptides and the oxidized type content is higher than that of reduced prototypes (Figure 3). The reason for the high glutathione content in quinoa may be due to the fact that quinoa contains more cysteine (about 5.5 mg/g protein) than plant-based proteins. Therefore, the consumption of quinoa-based foods can have a high nutritional value for children’s growth and development [21].

It is reported that polyphenols and saponins are the most important antioxidants in quinoa seeds [22,23]. The phenolics in quinoa seeds may be present in free form in vacuoles or in bound form on cell wall structures [24]. It has been found that there were significant differences in the content of bound phenolics among different quinoa cultivars, while their content of free phenolics showed relatively small differences [25,26]. At the same time, some studies have reported that bound phenolics were more responsible for the ability of scavenging DPPH and ABTS free radicals than free phenolics [27]. However, in this study, there was no general regularity between the contents of the bound phenolics and free phenolics in different cultivars, and the antioxidant properties of the phenolics depended on the free phenolics. Caffeic acid, syringic acid and ferulic acid were the main phenolic acids in quinoa (Table 1). Flavonoids are a class of antioxidant substances that account for a large proportion of phenolic substances [25], and had a high level in quinoa (Figure 2). Isorhamnetin is reported to be a flavonoid widely found in fruits such as ginkgo and sea buckthorn [28], which has multiple pharmacological activities, such as anti-inflammatory, antioxidant, and antitumor ones [29,30]. However, this study showed that quinoa also contains high level of isorhamnetin (Figure 4). Quercetin classes (Isoquercitin and quercetin) were also found to be the main substance of flavonoids in quinoa and their presence can increase the physiological activity of quinoa seeds [31]. Pasko et al. [32] analyzed the effects of the dietary addition of quinoa seeds on plasma and oxidative stress, and found that quinoa can improve antioxidant capacity by decreasing malondialdehyde in plasma and increasing the activity of antioxidant enzymes, which is consistent with our findings, proving that quinoa is a natural source of antioxidants.

Saponins are made up of steroids (or triterpenoids) and individual or multiple sugar chains. Saponins are often considered as anti-nutritional factors due to their effect on the digestibility and flavor profile of plant grains. However, saponins have been proved to have positive effects on the human body, such as antifungal, antioxidant and antiviral ones [33]. Stuardo and San [34] reported that the content of saponins in quinoa is between 0.1–5%, which is higher than that in beans and cereals. Phytolaccagenic acids had the highest levels among saponin monomers in the present research, which is consistent with the study of Lim et al. [22]. Lim et al. also reported that not only quinoa seeds, but also the roots and leaves of quinoa contain saponins, which are also potentially excellent raw materials for the development of functional substances [22]. Madl et al. [35] isolated 19 kinds of known saponins and 68 kinds of novel saponin compounds from the crude extract of triterpene saponins from quinoa seeds. The saponin compounds identified in this study were consistent with this.

Nucleosides are important metabolites in plants, which are not only important synthetic precursors of nucleotides, but also play an important role in plant growth as signaling molecules [36,37]. Although the composition of each nucleoside monomer was similar among different cultivars, the content varied, with the most significant differences were observed in the SJ-1 cultivars (Figure 6). Guanosine was the most abundant nucleoside compound in quinoa in this study. It has been reported that different types of guanosine play a key role in the metabolic pathways of different substances in the grain [38]. For example, cyclic guanosine monophosphate (cGMP) induces chalcone synthase (CHS) expression in photosignal transduction to indirectly participate in the synthesis of flavonoids [39]. Different kinds of small-molecule metabolites in plant grains are interconnected and play important nutritional roles for humans.

## 4. Materials and Methods

### 4.1. Raw Materials

Five cultivars of quinoa (Qingli-1, QL-1; Sanjiang-1, SJ-1; Sanjiang-2, SJ-2; Keli-1, KL-1 and Keli-2, KL-2) used in this study were harvested in Qinghai province in 2021 and provided by Qinghai Academy of Agricultural and Forestry Sciences. The dried seeds were stored at −20 °C before use.

### 4.2. Chemicals and Reagents

LC–MS-grade methanol (MeOH), acetonitrile (ACN), and methyl tert-butyl ether (MTBE) were purchased from VWR International (Zaventem, Belgium). Ammonium formate (99%) was obtained from Sigma-Aldrich (St. Louis, MO, USA). Ultrafree^®^-MC centrifugal filter devices (0.22 μm) were obtained from Millipore (Bredford, MA, USA). DPPH and ABTS were purchased from Yuanye Bio-Technology Co., Ltd. (Shanghai, China). Other chemicals and reagents were of analytical grade.

### 4.3. Metabolomic Analysis

Metabolomic analysis were determined according to the literature, with slight modifications [40]. The freeze-dried quinoa sample was ground into powder and sieved through an 80-micron-mesh sieve (mesh size: 180 µm). Twenty milligrams of powder was mixed with 200 μL methanol by shaking for 10 min and centrifuged (15,000× *g*) at 4 °C for 10 min. The supernatant was filtered through a syringe filter (0.22 μm, organic phase) and we waited for analysis with a UPLC-ZenoTOF-MS/MS system. To prepare the QC sample, 20 μL of all sample extracts were mixed by vortex and the QC sample was injected once every five samples to detect instrument drift.

Liquid chromatography was performed by an ExionLC AD ultra-performance liquid chromatography (UPLC, AB Sciex Analytical Instrument Trading Co., Ltd., Shanghai, China) system. The analytical column was a Waters ACQUITY UPLC HSS T3 (1002.1 mm, 1.8 μm; Waters, Zellik, Belgium), with a flow rate set to 0.4 mL/min and an injection volume of 3 μL. Mobile phase A and B were 0.05% formic acid and acetonitrile, respectively. The column and sample temperature were maintained at 40 °C. A gradient elution program was started with 1% B, and from 1 to 24 min, it linearly increased to 30% B. From 24 to 37 min, it increased to 95% B and was maintained for 3 min, and it decreased to 1% at 44 min.

The UPLC system was coupled to a ZenoTOF7600 mass spectrometer equipped with an electrospray ionization (ESI) source (AB Sciex, Shanghai, China). The ESI source conditions were set as follows: spray voltage (5.5 kV), curtain gas (N_2_, 99.999%, 35 psi, Domnik Hunter), temperature (550 °C), ion source gas1 (nebulizer gas, 55 psi), and ion source gas 2 (turbo gas, 55 psi). Data were collected in both positive and negative ionization modes over a mass range between 60 and 1200 *m/z*. The MS/MS experiments were performed using a collision energy of 35 eV in negative mode. The mass range was set between 40 and 1200 *m/z* for the fragmentation products.

The acquisition of the raw data was performed using Analyst TF (version 1.7.1, AB Sciex, Framingham, MA, USA) qualitative analysis software. The data processing was performed in the online tool Metaboanalyst 5.0 (https://www.metaboanalyst.ca/; accessed on 12 June 2023), with processes including peak picking, quality assurance, normalization (by median), log transformation (base 10), and auto scaling (mean-centered and divided by the standard deviation of each variable). The molecular weights of the metabolites were confirmed, matched, and annotated by databases (Metlin, MassBank and KNApSAcK databases) to achieve accurate metabolite characterization.

### 4.4. Determination of Total Phenolic Content

#### 4.4.1. Extraction

The extraction and the determination of the total phenolic content in quinoa was conducted according to a method in the literature [41], with slight modifications. Briefly, 1 g quinoa powder was mixed with 20 mL of 80% methanol and extracted in the darkness at 25 °C with shaking (200 rpm) for 1 h. Then, mixtures were centrifuged (10,000× *g*, 15 min) and the supernatants from three cycles of centrifugations were evaporated to dry at 40 °C. The dried samples were re-dissolved by 50% methanol and stored at −20 °C before analysis as a free phenolic extract (FPS).

The residues after extraction of the free phenolic were mixed with 20 mL of 2 mol/L NaOH to reach a pH between 1.5 and 2.0. After being oscillated at 25 °C in the absence of light for 4 h, the mixture was mixed with 25 mL of ethyl acetate for 15 min and centrifuged (10,000× *g*, 15 min) to collect the ethyl acetate layer. Ethyl acetate layers from 3 cycles of centrifugation were combined and dried in a rotary evaporator at 40 °C. The residue was re-dissolved into 10 mL of 50% methanol as a bound phenolic solution (BPS) and stored at −20 °C before analysis.

#### 4.4.2. Determination of Contents of Total Phenolic and Phenolic Acids

The content of the total phenolics was determined by Folin’s phenol method, according to our previous study [42]. Galic acid was used as a standard and the absorbance at 765 nm was measured with a 50% methanol solution used as the blank control. The total phenolic content was presented in mg GAE/100 g DW.

The quantification of phenolic acids was conducted using high-performance liquid chromatography (HPLC, Agilent Technologies Co., Ltd., Santa Clara, CA, USA) as described by the literature [43], with slight modification. FPS and BPS were filtered through a 0.45 μm membrane filter for the Agilent 1200 HPLC system with a reversed phase column (Shimadzu C18 110A, Kyoto, Japan, 4.6 mm × 150 mm, 5 μm particle size) and a C18 cartridge. The column temperature was maintained at 35 °C. Mobile phase A and B were water and methanol; both contained 0.1% acetic acid, respectively. A 75 min gradient was programmed as follows: 0–11 min, 9–14% B; 11–14 min, 14–15% B; 14–17 min, 15% B; 17–24 min, 15–16.5% B; 24–28 min, 16.5–19% B; 28–30 min, 19–25% B; 30–36 min, 25–26% B; 36–38 min, 26–28% B; 38–41 min, 28–35% B; 41–46 min, 35–40% B; 46–48 min, 40–48% B; 48–53 min, 48–53% B; 53–65 min, 53–70% B; 65–66 min, 70–9% B; 66–75 min, 9% B. The injection volume was 20 μL and the flow rate was 0.9 mL/min. The detector was VWD and the measurement wavelength was 280 nm.

### 4.5. Determination of ABTS and DPPH Radical-Scavenging Activities

The ABTS and DPPH radical-scavenging activities of different cultivars of quinoa were measured as described in the literature [43]. The ABTS radical cation (ABTS^+^) was created by reacting a 7.0 mM stock solution of ABTS (20 mL) with 13.2 mg potassium persulphate, and the mixture was stirred in the dark at room temperature (25 °C) for 16 h. The ABTS^+^ solution was diluted with PBS buffer (pH 7.4) with an absorbance of 0.7 ± 0.1 at 734 nm. The absorbance was measured at 734 nm after mixing a certain amount of diluted FPS and BPS with ABTS working solution. The DPPH working solution (0.05 mM) was prepared. FPS and BPS were diluted and mixed with DPPH working solution for 30 min. A UV–visible spectrophotometer was used to measure the absorbance of the mixture at 517 nm. Trolox was used as the standard. The results were expressed as micromoles of Trolox equivalents per gram of dry sample (μ molTE/DW).

### 4.6. Statistical Analysis

All measurements were taken at least three times. Metabolomic data analysis, i.e., PCA/PLS-DA, was carried out using the online tool Metaboanalyst 5.0, according to the provided protocols in the website (https://www.metaboanalyst.ca/; accessed on 12 June 2023). The data were presented as means ± SD from at least three replicates. GraphPad Prism 7 was used for data elaboration, and one-way ANOVA as well as Duncan’s test were used for statistical analysis. Statistical significance was established at *p* < 0.05.

## 5. Conclusions

In the present study, a total of 248 metabolites from five cultivars of quinoa were identified and compared. Significant variations in the profiles of phenolics, amino acids, saponins and nucleosides among the different quinoa cultivars were observed. SJ-1 exhibited a unique metabolite profile among the five cultivars due to its composition of amino acids/peptides and nucleosides. In addition, the antioxidant capacities of the quinoas was found to be closely related to their free phenolic acid contents. These findings will provide useful information for developing functional foods with quinoa and explore the potential of this crop.

## Figures and Tables

**Table 1:** The content of individual phenolic acids in different cultivars of quinoa.

Form	Cultivars	Phenolic Acid Content (mg/kg)								
		Gallic Acid	Protocatechuic Acid	*p*-Hydroxybenzoic Acid	Vanillic Acid	Caffeic Acid	Syringic Acid	*p*-Coumaric Acid	Ferulic Acid	Sinapinic Acid
Bound	QL-1	4.34 ± 0.46 ^c^	ND	ND	ND	7.39 ± 1.62 ^b^	3.82 ± 0.15 ^c^	0.30 ± 0.03 ^b^	156.11 ± 3.95 ^a^	0.32 ± 0.04 ^c^
	SJ-1	9.93 ± 0.87 ^a^	ND	ND	ND	6.87 ± 1.62 ^b^	35.75 ± 1.76 ^a^	0.25 ± 0.02 ^b^	153.86 ± 4.65 ^a^	0.48 ± 0.03 ^b^
	SJ-2	9.20 ± 1.39 ^a^	ND	ND	ND	9.73 ± 2.04 ^a^	12.28 ± 1.62 ^b^	0.24 ± 0.03 ^b^	136.66 ± 14.04 ^b^	0.50 ± 0.03 ^b^
	KL-1	5.14 ± 1.20 ^b^	ND	ND	0.84 ± 0.44 ^a^	9.46 ± 2.62 ^a^	2.72 ± 0.56 ^c^	0.56 ± 0.05 ^a^	128.08 ± 16.67 ^b^	0.69 ± 0.08 ^a^
	KL-2	4.24 ± 1.15 ^c^	ND	0.86 ± 0.39 ^a^	0.98 ± 0.38 ^a^	5.18 ± 2.63 ^b^	3.09 ± 1.13 ^c^	0.48 ± 0.06 ^a^	132.44 ± 19.17 ^b^	0.55 ± 0.12 ^b^
Free	QL-1	4.11 ± 0.40 ^d^	0.03 ± 0.04 ^a^	ND	ND	23.85 ± 0.42 ^b^	ND	0.12 ± 0.01 ^b^	ND	0.19 ± 0.01 ^b^
	SJ-1	13.19 ± 0.34 ^a^	0.01 ± 0.00 ^a^	ND	ND	32.38 ± 3.10 ^a^	3.42 ± 0.14 ^a^	0.18 ± 0.01 ^a^	69.96 ± 6.79 ^a^	0.15 ± 0.01 ^c^
	SJ-2	6.69 ± 0.60 ^b^	0.01 ± 0.00 ^a^	ND	ND	21.91 ± 2.24 ^b^	1.37 ± 0.57 ^b^	0.08 ± 0.01 ^c^	25.79 ± 6.20 ^b^	0.11 ± 0.02 ^d^
	KL-1	7.29 ± 0.55 ^b^	0.01 ± 0.00 ^a^	ND	ND	24.30 ± 1.02 ^b^	ND	0.18 ± 0.01 ^a^	ND	0.27 ± 0.06 ^a^
	KL-2	5.66 ± 0.10 ^c^	0.01 ± 0.00 ^a^	ND	ND	24.18 ± 1.21 ^b^	ND	0.17 ± 0.01 ^a^	ND	0.28 ± 0.02 ^a^

Note: ND represents that there is no detection. Different letters in same column represent significant difference between different cultivars of quinoa (*p* < 0.05).

## Data Availability

Data are contained within the article.

## Supplementary Materials

The following supporting information can be downloaded at: https://www.mdpi.com/article/10.3390/plants13020240/s1.
