# Supplementary material for: Metabolomics Analysis of Different Quinoa Cultivars Based on UPLC-ZenoTOF-MS/MS and Investigation into Their Antioxidant Characteristics
Source: Plants (Basel). 2024 Jan 15;13(2):240. doi: 10.3390/plants13020240 (PMC10819959; doi:10.3390/plants13020240)
Supplement: Supplementary file 1 [file plants-13-00240-s001.zip › plants-2795861-supplementary.pdf]

plants

Supplementary Material for

Metabolomics Analysis of Different Quinoa Cultivars Based on UPLC-ZenoTOF-MS/MS and Investigation into Their Antioxidant Characteristics

Shufang Wang [1,2], Guannan Liu [1], Chong Xie [1], You Zhou [2], Runqiang Yang [1], Jirong Wu [2], Jianhong Xu [1,2,*] and Kang Tu [1,*]

[1] College of Food Science and Technology, Whole Grain Food Engineering Research Center, Nanjing Agricultural University, Nanjing 210095, China; wangshufang202301@163.com (S.W.); 2022208029@stu.njau.edu.cn (G.L.); xiechong@njau.edu.cn (C.X.); yangrq@njau.edu.cn (R.Y.)

[2] Jiangsu Key Laboratory for Food Quality and Safety-State Key Laboratory Cultivation Base, Ministry of Science and Technology/Key Laboratory for Control Technology and Standard for Agro-Product Safety and Quality, Ministry of Agriculture and Rural Affairs/Key Laboratory for Agro-Product Safety Risk Evaluation (Nanjing), Ministry of Agriculture and Rural Affairs/Collaborative Innovation Center for Modern Grain Circulation and Safety/Institute of Food Safety and Nutrition, Jiangsu Academy of Agricultural Sciences, Nanjing 210014, China; zhouyouoox@163.com (Y.Z.); yangzhouwjr@126.com (J.W.)

* Correspondence: xjh@jaas.ac.cn (J.X.); kangtu@njau.edu.cn (K.T.); Tel.: +86-025-84392001 (J.X.); Fax: +86-025-84399016 (K.T.)

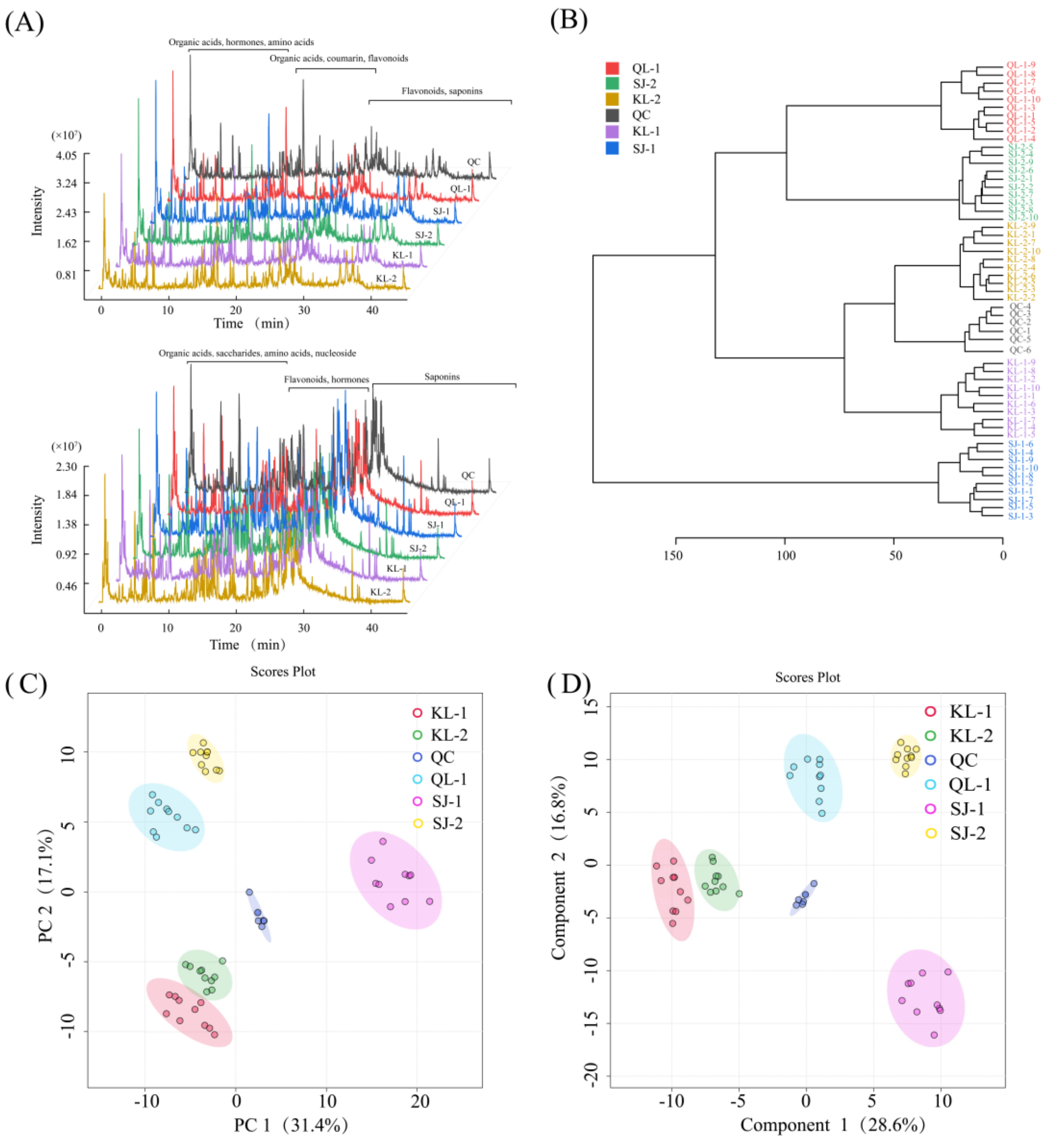

Figure S1. Isolation and identification of metabolites in different cultivars of quinoa. Total ion chromatogram of metabolite molecular species from different cultivars of quinoa under positive (Upper one) and negative (Lower one) ion mode (A). Hierarchical cluster dendritic diagram from different cultivars of quinoa under positive and negative ion mode (B). PCA score plot (C) and PLS-DA score plot (D) of identified metabolites in different cultivars of quinoa.

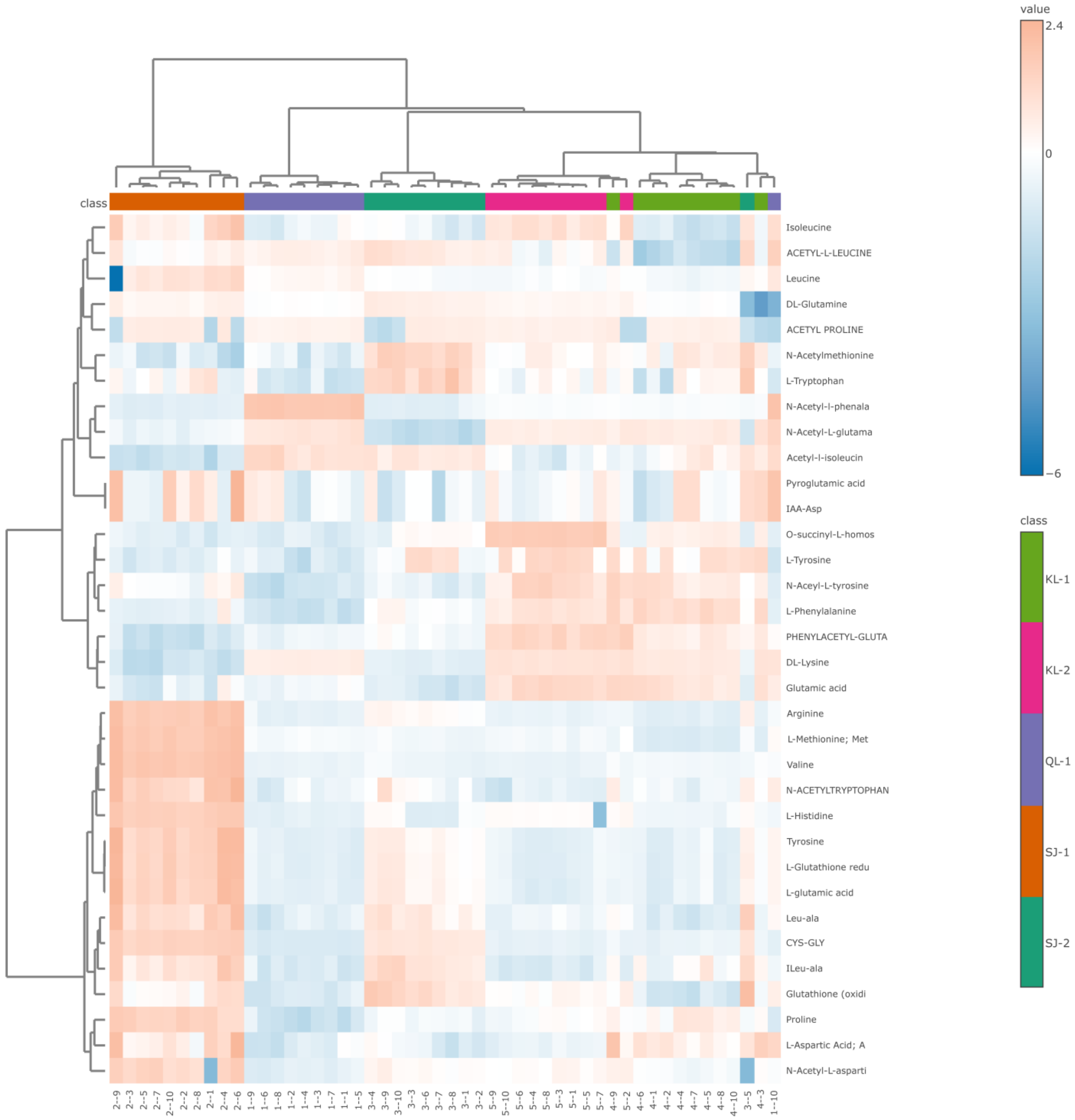

Fig S2 Heatmaps based on top 25 amino acids/peptides in different varieties of quinoa. Colors represent different concentrations indicated by the color bar.

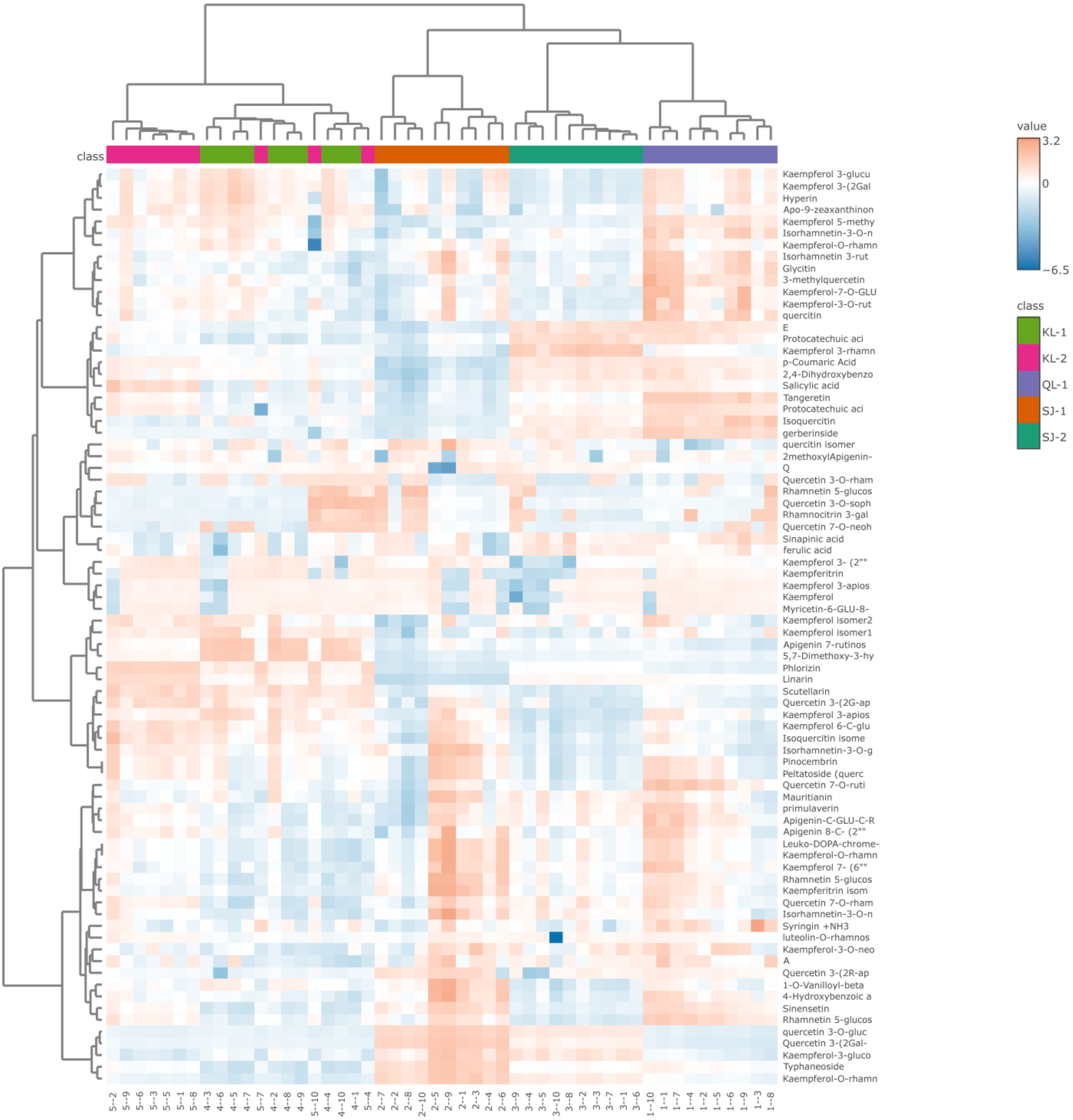

Fig S3 Heatmaps based on top 25 phenolic acids in different varieties of quinoa. Colors represent different concentrations indicated by the color bar.

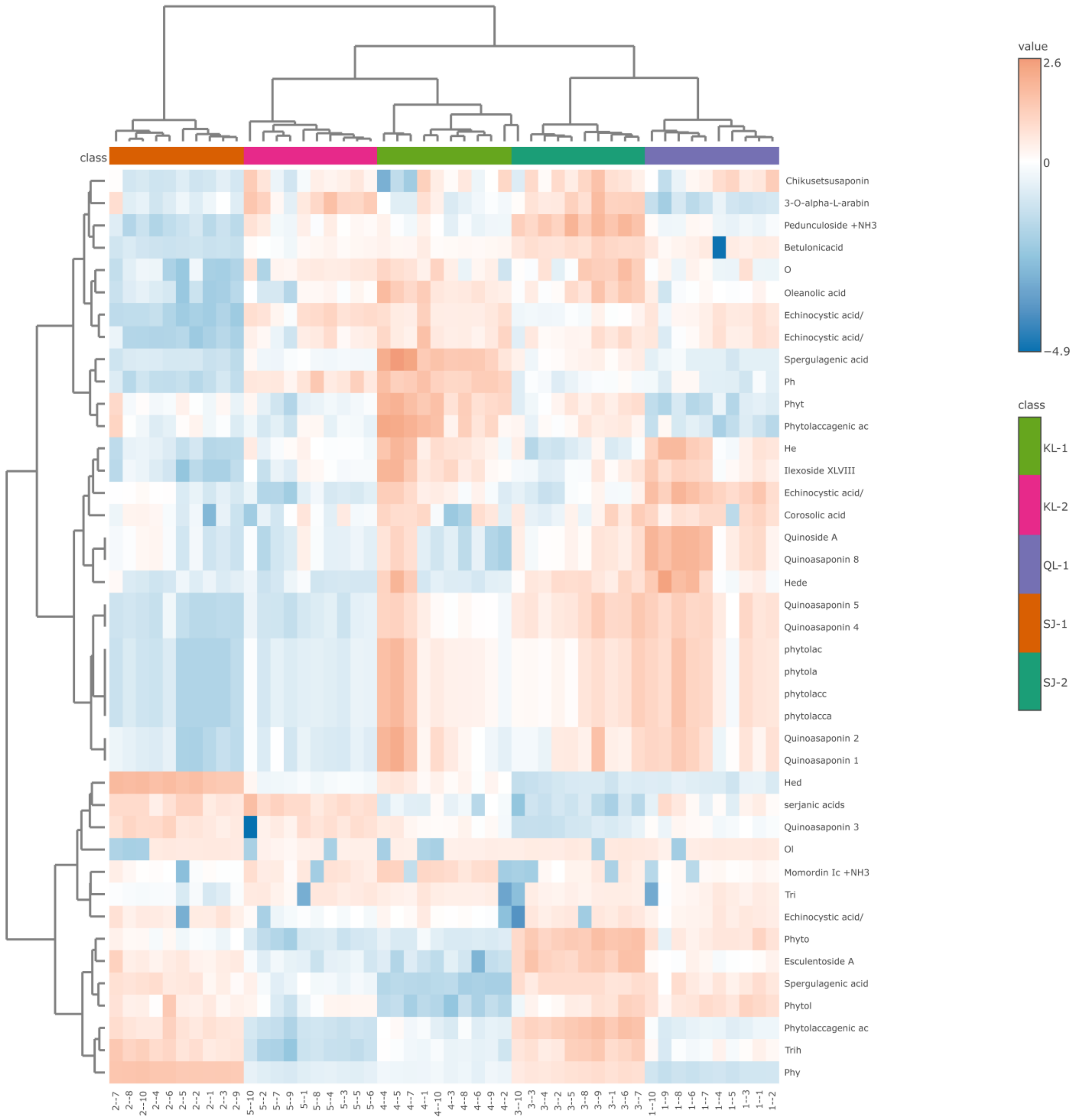

Fig S4 Heatmaps based on top 25 saponins in different varieties of quinoa. Colors represent different concentrations indicated by the color bar.

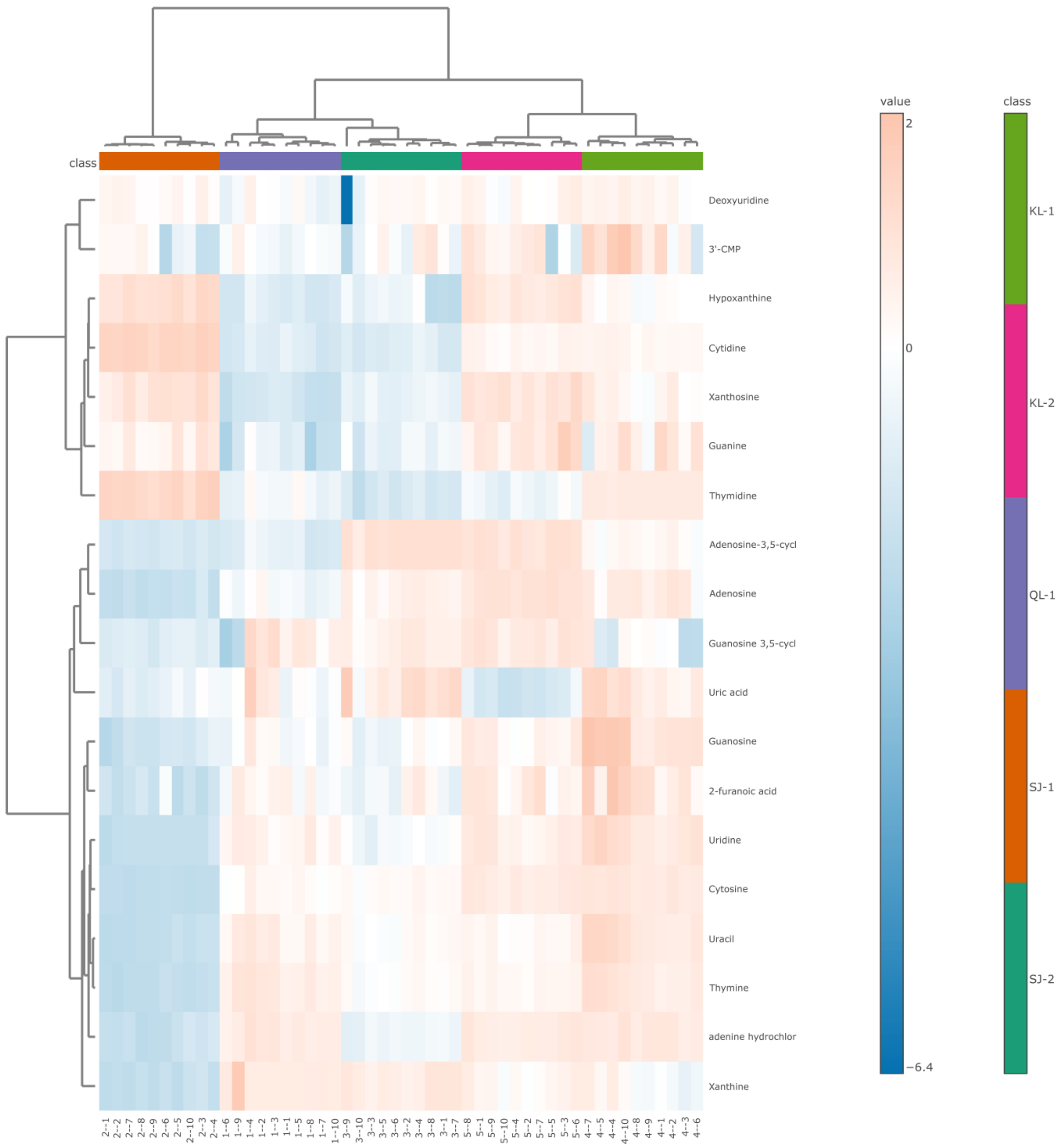

Fig S5 Heatmaps based on top 25 nucleosides in different varieties of quinoa. Colors represent different concentrations indicated by the color bar.

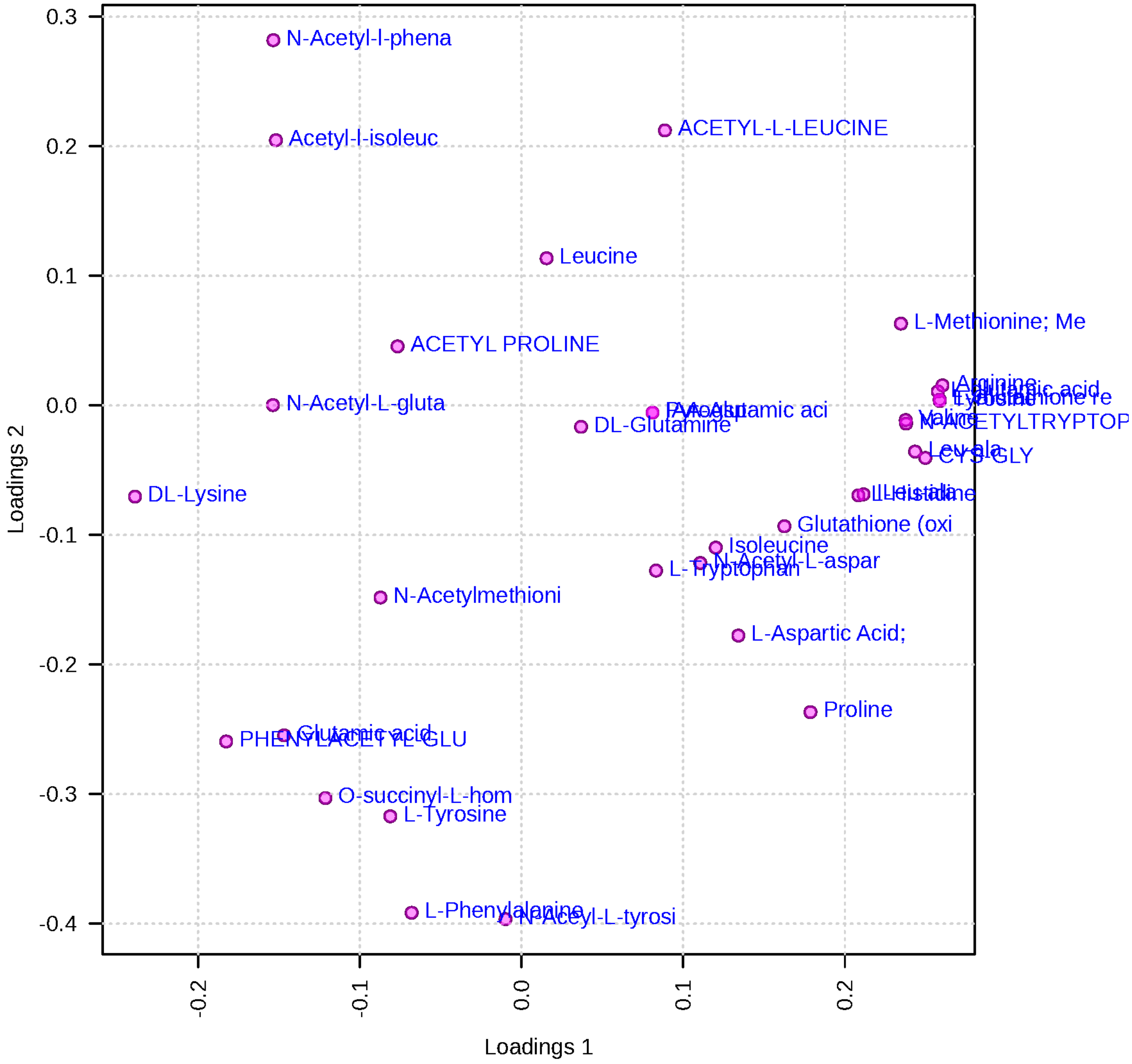

Fig S6 Loading plots of amino acids/peptides in different varieties of quinoa.

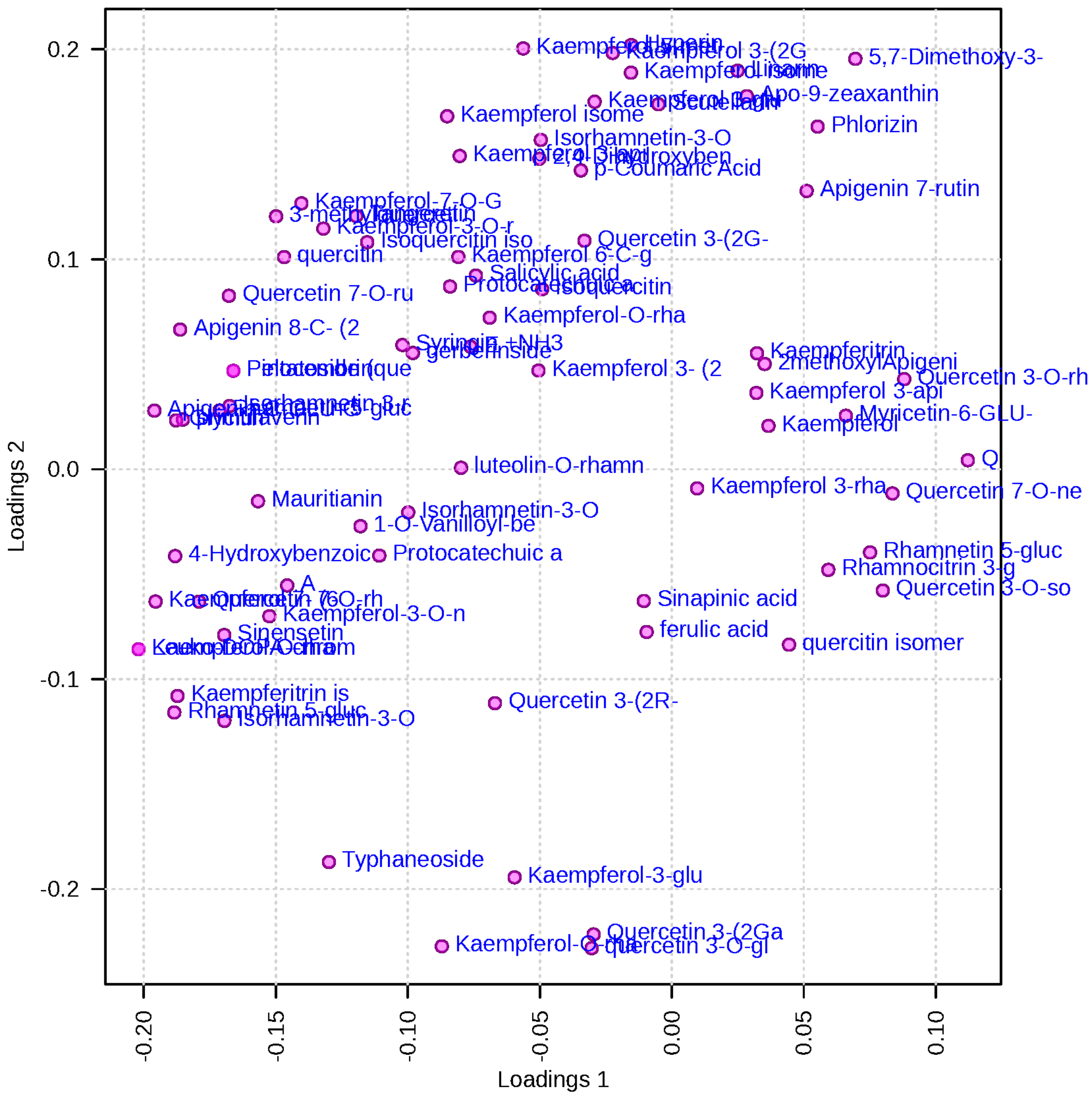

Fig S7 Loading plots of phenolic acids in different varieties of quinoa.

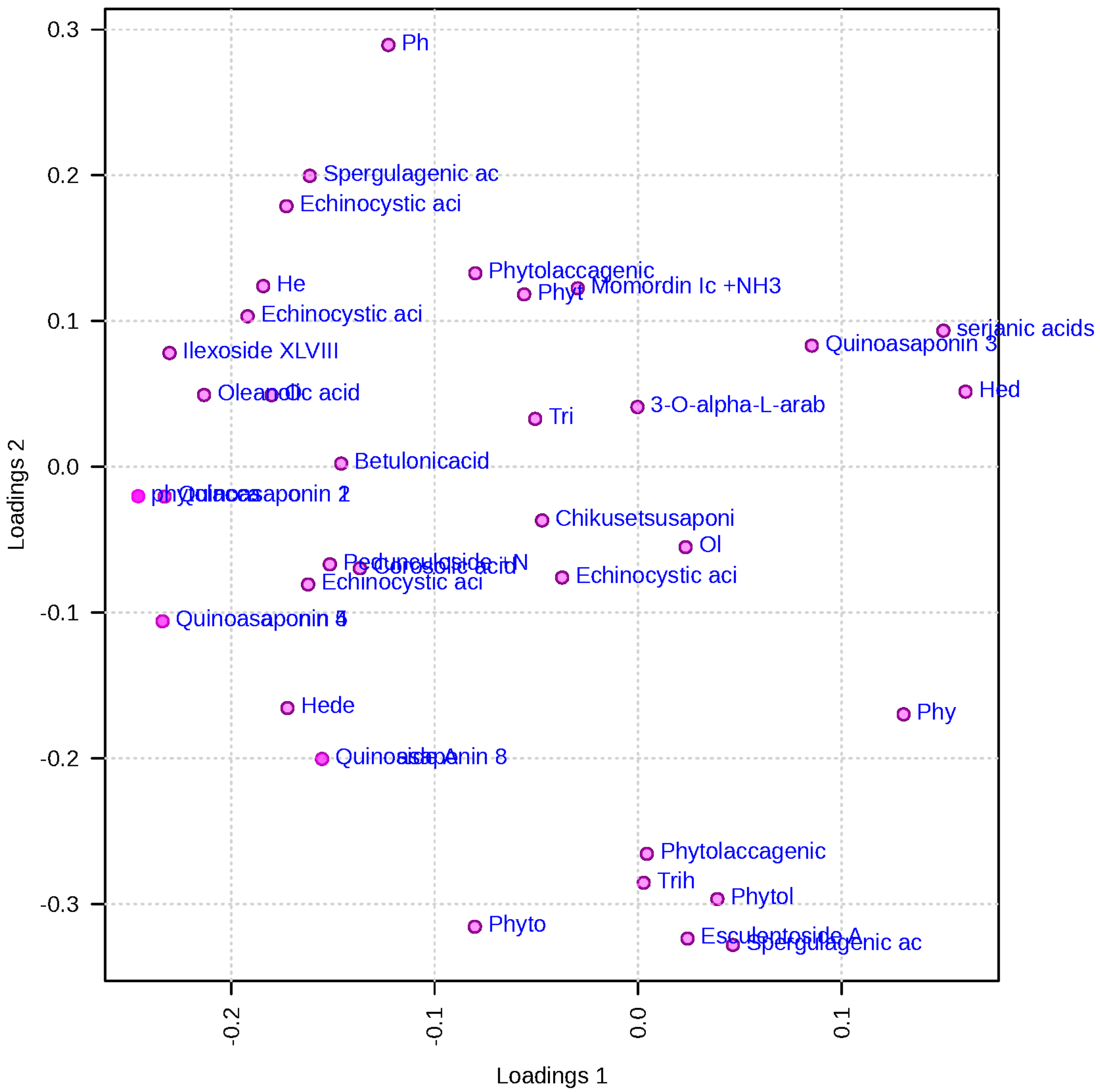

Fig S8 Loading plots of saponins in different varieties of quinoa.

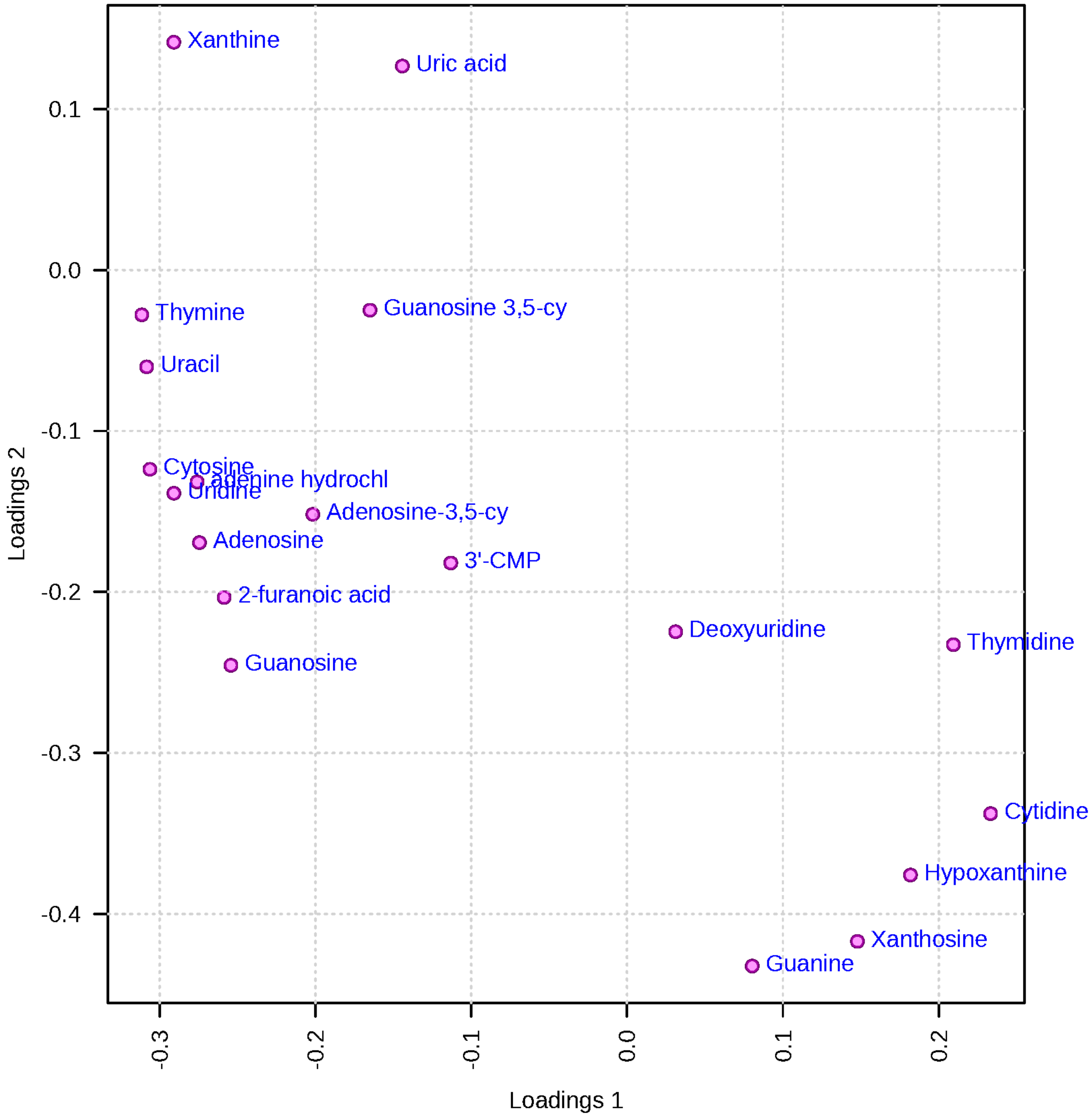

Fig S9 Loading plots of amino nucleosides in different varieties of quinoa.

Tab S1 Types of metabolites detected in different varieties of quinoa.

Copyright: © 2024 by the authors. Licensee MDPI, Basel, Switzerland. This article is an open access article distributed under the terms and conditions of the Creative Commons Attribution (CC BY) license (https://creativecommons.org/licenses/by/4.0/).

Metabolite type	Metabolites
Amino acid/peptide	ACETYL PROLINE
	Acetyl-l-isoleucine
	ACETYL-L-LEUCINE
	CYS-GLY
	DL-Glutamine
	DL-Lysine
	Glutamic acid
	Glutathione (oxidized)
	IAA-Asp
	ILeu-ala
	Isoleucine
	L-Aspartic Acid; Asp
	Leu-ala
	Leucine
	L-Glutathione reduced
	L-Histidine
	L-Methionine; Met H -H
	L-Phenylalanine
	L-Tryptophan
	L-Tyrosine
	N-Acetyl-L-aspartic acid
	N-Acetyl-L-glutamate
	N-Acetyl-l-phenalanine
	N-Acetylmethionine
	N-ACETYLTRYPTOPHAN
	N-Aceyl-L-tyrosine
	O-succinyl-L-homoserine
	PHENYLACETYL-GLUTAMINE
	Proline
	Pyroglutamic acid
	Tyrosine
	Valine
	Arginine

	L-glutamic acid
Sesquiterpene	Curdione
	Patchouli alcohol (loss H20)
Nucleoside	2-furanoic acid
	3'-CMP
	adenine hydrochloride
	Adenosine
	Adenosine-3,5-cyclic phosphate cAMP
	Cytidine
	Cytosine
	Deoxyuridine
	Guanine
	Guanosine
	Guanosine 3,5-cyclic monophosphate
	Hypoxanthine
	Thymidine
	Thymine
	Uracil
	Uric acid
	Uridine
	Xanthine
	Xanthosine
Phenolics	2,4-Dihydroxybenzoic acid
	4-Hydroxybenzoic acid
	ferulic acid
	p-Coumaric Acid
	Protocatechuic acid
	Protocatechuic acid-3-glucoside
	Salicylic acid
	Sinapinic acid
	1-O-Vanilloyl-beta-D-glucose
	E
	2methoxylApigenin-O-GLU-O-Rha
	3-methylquercetin
	A
	5,7-Dimethoxy-3-hydroxyflavone
	Apigenin 7-rutinoside
	Apigenin 8-C- (2",3"-diacetylglucoside)
	Apigenin-C-GLU-C-Rha
	Apo-9-zeaxanthinone
	gerberinside
	Glycitin

	Hyperin
	Isoquercitin
	Isoquercitin isomer
	Isorhamnetin 3-rutinoside (Narcissoside)
	Isorhamnetin-3-O-glucoside
	Isorhamnetin-3-O-neohespeidoside
	Isorhamnetin-3-O-neohespeidoside isomer
	Kaempferitrin
	Kaempferitrin isomer
	Kaempferol
	Kaempferol 3- (2''- (E) -p-coumaroyl-alpha-L-arabinofuranoside) -8-C-rhamnoside
	Kaempferol 3-(2Gal-apiosylrobinobioside)
	Kaempferol 3-apiosyl-(1->2)-galactoside
	Kaempferol 3-apiosyl-(1->4)-rhamnoside-7-rhamnoside
	Kaempferol 3-glucuronide
	Kaempferol 3-rhamnoside-7-xyloside
	Kaempferol 5-methyl ether
	Kaempferol 6-C-glucoside-8-C-rhamnoside
	Kaempferol 7- (6''-p-succinylglucoside)
	Kaempferol isomer1
	Kaempferol isomer2
	Kaempferol-3-glucoside (Astragalin)
	Kaempferol-3-O-neohespeidoside
	Kaempferol-3-O-rutinoside
	Kaempferol-7-O-GLU
	Kaempferol-O-rhamnoside isomer1
	Kaempferol-O-rhamnoside isomer2
	Kaempferol-O-rhamnoside isomer3
	Leuko-DOPA-chrome-glucoside
	Linarin
	luteolin-O-rhamnoside-GLU
	Mauritianin
	Myricetin-6-GLU-8-Xyl

	Peltatoside (quercetin-3-arabinoglucoside)<br>Phlorizin<br>Pinocembrin<br>primulaverin<br>Quercetin 3-(2Gal-rhamnosyl-robinobioside)<br>Quercetin 3-(2G-apiosylrutinoside)<br>Quercetin 3-(2R-apiosylrutinoside)<br>quercetin 3-O-glucuronide<br>Quercetin 3-O-rhamnosyl-(1->6)-galactoside<br>Quercetin 3-O-sophoroside<br>Quercetin 7-O-neohespeidoside<br>Quercetin 7-O-rhamnosyl-(1->6)-galactoside<br>Quercetin 7-O-rutinoside<br>Q<br>quercitin<br>quercitin isomer<br>Rhamnetin 5-glucoside<br>Rhamnetin 5-glucoside isomer1<br>Rhamnetin 5-glucoside isomer2<br>Rhamnocitrin 3-galactoside<br>Scutellarin<br>Sinensetin<br>Syringin +NH3<br>Tangeretin<br>Typhaneoside
hormone	Corticosterone<br>ecdysterone<br>Indole<br>Norethisterone acetate
pigment	4,5-seco-DOPA<br>Amaranthin<br>Betanin<br>DOPA-betaxanthin
alkaloid	N-trans-Feruloyl tyramine<br>Sinapine<br>Trigonelline
sugar	D-Glucose<br>D-Sucrose

	nystose<br>RAFFINOSE<br>Sibiricose A1<br>Sibiricose A5<br>Stachyose<br>D-Glucose 1-phosphate<br>Fungitetraose<br>Po<br>Pom
vitamin	4-Pyridoxic Acid<br>Nicotinic acid<br>Vitamin B2<br>Vitamin B4<br>Vitamin B5
coumarin	6-Methylcoumarin<br>Cinnamaldehyde<br>Coumarin<br>Scoparone
organic acid	2-Aminoadipic acid<br>2-ISOPROPYLMALIC ACID<br>3-(4-Hydroxyphenyl)lactate<br>anthranilic acid<br>CITRIC ACID<br>DL-3-Aminoisobutyric acid<br>DL-Malic acid -H<br>glutaconic acid<br>hexadecylitaconic acid<br>Hexaric acid<br>L-Threonic acid<br>methylene succinic acid<br>Pantothenic Acid<br>Pipecolic acid<br>Pipecolinic acid<br>Succinic acid<br>trans-3-indole-acrylic acid<br>Vanillic acid<br>Glycyrrhetinic acid
saponin	3-O-alpha-L-arabinopyranosylserjanic acid 28-O-beta-D-glucopyranosyl ester<br>3β,23,30 trihydroxy olean-12-en-28-oic acid

	3β,23,30 trihydroxy olean-12-en-28-oic acid isomer<br>Betulonicacid<br>Chikusetsusaponin IVa<br>Corosolic acid<br>Echinocystic acid/Hederagenin/Queretaroic acid 1<br>Echinocystic acid/Hederagenin/Queretaroic acid 2<br>Echinocystic acid/Hederagenin/Queretaroic acid 3<br>Echinocystic acid/Hederagenin/Queretaroic acid 4<br>Esculentoside A<br>Ilexoside XLVIII<br>Momordin Ic +NH3<br>Oleanolic acid<br>oleanolic acid 3-O-[α-L-aαabinopyαanosyl-(1-3)-βD-glucuαonopyαanosyl]-28-O-β-D-glucopyαanoside<br>oleanolic acid 3-O-β-D-glucopyαanosyl-28-O-β-D-glucopyαanoside<br>Pedunculoside +NH3<br>Phytolaccagenic acid<br>phytolaccagenic acid 3-O- [α-L-arabinopyranosyl-(1-3)-β-D-glucuronopyranosyl]- 28-O-β-D-glucopyranoside<br>phytolaccagenic acid 3-O- [α-L-arabinopyranosyl-(1-3)-β-D-glucuronopyranosyl]- 28-O-β-D-glucopyranoside isomer<br>phytolaccagenic acid 3-O-[β-D-glucopyranosyl-(1-4)-β-D-glucopyranosyl-(1-4)-β-D-glucopyranosyl]-28-O-β-D-glucopyranoside<br>phytolaccagenic acid 3-O-[β-D-glucopyαanosyl-(1-4)-β-D-glucopyαanosyl]-28-O-β-D-glucopyαanoside

	phytolaccagenic acid 3-O-[β-D-glucopyαanosyl-(1-4)-β-D-glucopyαanosyl]-28-O-β-D-glucopyαanoside isomer
	Phytolaccagenic acid isomer
	phytolaccagenic acid-3-O-β-D-glucopyαanosyl-28-O-β-D-glucopyαanoside
	phytolaccagenic acid-3-O-β-D-glucopyαanosyl-28-O-β-D-glucopyαanoside isomer1
	phytolaccagenic acid-3-O-β-D-glucopyαanosyl-28-O-β-D-glucopyαanoside isomer2
	phytolaccagenic acid-3-O-β-D-glucopyαanosyl-28-O-β-D-glucopyαanoside isomer3
	Quinoasaponin 1
	Quinoasaponin 2
	Quinoasaponin 3
	Quinoasaponin 4
	Quinoasaponin 5
	Quinoasaponin 8
	Quinoside A
	serjanic acids
	spergulagenic acid 3-O-[α-L-arabinopyranosyl-(1-3)- β-D-glucuronopyranosyl]-28-O-β-D-glucopyranoside
	hedeαagenin 3-O-[α-L-aαabinopyαanosyl- (1-3)-β-D-glucuαonopyαanosyl]-28-O-β-D-glucopyαanoside
	hedeαagenin 3-O-[α-L-aαabinopyαanosyl- (1-3)-β-D-glucuαonopyαanosyl]-28-O-β-D-glucopyαanoside isomer1
	hedeαagenin 3-O-[α-L-aαabinopyαanosyl- (1-3)-β-D-glucuαonopyαanosyl]-28-O-β-D-glucopyαanoside isomer2

	spergulagenic acid 3-O-[β-D-glucopyranosyl-(1-2)-β-D-glucopyranosyl-(1-3)- α-L-arabinopyranosyl]-28-O-β-D-glucopyranoside
other	(Z)-3-[4-methoxy-2-[(2S,3R,4S,5S,6R)-3,4,5-trihydroxy-6-(hydroxymethyl)oxan-2-yl]oxyphenyl]prop-2-enoic acid<br>13-Docosenamide<br>13-Oxo-9,11-tridecadienoic acid<br>1-amino-naphthalene<br>1-O-Vanilloyl-beta-D-Galactose<br>2,3-dihydro-3,5-dihydroxy-6-methyl-4H-pyran-4-one<br>3-hydroxytyramine hydrochloride<br>4-hydroxy-6-methyl-2-pyron<br>7-hydroxy-4-methyl-chromen-2-one<br>9-(2,3-dihydroxypropoxy)-9-oxononanoic acid<br>Acetophenone<br>C18:2-glycer-Glu<br>Choline<br>ethyl 6-(4-hydroxyphenyl)-4-methyl-2-oxo-1,3,6-trihydropyrimidine-5-carboxylat e<br>Gingerglycolipid B<br>Levodopa<br>Methyl linoleate<br>N-trans-Isoferuloyl-3-O-methyldopamine<br>Phosphocholine<br>pinellic acid<br>piperidine<br>Momordin IIc<br>Sambucinol H -H<br>Methoxsalen

Disclaimer/Publisher's Note: The statements, opinions and data contained in all publications are solely those of the individual author(s) and contributor(s) and not of MDPI and/or the editor(s). MDPI and/or the editor(s) disclaim responsibility for any injury to people or property resulting from any ideas, methods, instructions or products referred to in the content.
